# Genome-Wide Identification, Expression Analysis and Functional Study of *CCT* Gene Family in *Medicago truncatula*

**DOI:** 10.3390/plants9040513

**Published:** 2020-04-16

**Authors:** Lin Ma, Dengxia Yi, Junfeng Yang, Xiqiang Liu, Yongzhen Pang

**Affiliations:** 1 Institute of Animal Science, Chinese Academy of Agricultural Sciences, Beijing 100193, China; malin@caas.cn (L.M.); yidengxia@caas.cn (D.Y.); jfyang63@ibcas.ac.cn (J.Y.); xiqiangliu003@126.com (X.L.); 2 Key Laboratory of Plant Resources and Beijing Botanical Garden, Institute of Botany, the Chinese Academy of Sciences, Beijing 100093, China; 3 Department of Grassland Science, China Agriculture University, Beijing 100193, China

**Keywords:** *Medicago truncatula*, flowering time, *CCT*, circadian rhythms, hormones, abiotic stress

## Abstract

The control of flowering time has an important impact on biomass and the environmental adaption of legumes. The *CCT* (*CO*, *COL* and *TOC1*) gene family was elucidated to participate in the molecular regulation of flowering in plants. We identified 36 *CCT* genes in the *M*. *truncatula* genome and they were classified into three distinct subfamilies, *PRR* (7), *COL* (11) and *CMF* (18). Synteny and phylogenetic analyses revealed that *CCT* genes occurred before the differentiation of monocot and dicot, and *CCT* orthologous genes might have diversified among plants. The diverse spatial-temporal expression profiles indicated that *MtCCT* genes could be key regulators in flowering time, as well as in the development of seeds and nodules in *M. truncatula*. Notably, 22 *MtCCT* genes with typical circadian rhythmic variations suggested their different responses to light. The response to various hormones of *MtCCT* genes demonstrated that they participate in plant growth and development via varied hormones dependent pathways. Moreover, six *MtCCT* genes were dramatically induced by salinity and dehydration treatments, illustrating their vital roles in the prevention of abiotic injury. Collectively, our study provides valuable information for the in-depth investigation of the molecular mechanism of flowering time in *M. truncatula*, and it also provides candidate genes for alfalfa molecular breeding with ideal flowering time.

## 1. Introduction

Flowering time is a crucial trait that determines plant regional adaptability to diverse environmental conditions, as well as the grain yield of cereal crops [1,2]. In legume forage crops, flowering time also serves as an indicator for harvesting, as famers often cut legumes at the early bloom stage. It is important to elucidate flowering time-related genes and their regulation mechanism in alfalfa and other legumes. Flowering time is affected by numerous environmental and endogenous signals, via intricate molecular pathways [3]. The molecular mechanism underlying flowering time has been extensively studied in model plants, and genes regulate the transition from vegetative to reproductive growth, mainly through photoperiod, vernalization, autonomous and gibberellin pathways [1]. Among them, *CCT* family members widely participate in the regulation of flowering time through photoperiod and vernalization pathways, and they also play vital roles in plant growth and development [4,5,6].

The *CCT* genes contain a CCT domain that originally corresponded to a 43-amino-acid sequence in the carboxy-terminus of three proteins in *Arabidopsis thaliana*, namely CO (CONSTANS), COL (CO-LIKE), and TOC1 (TIMING OF CAB1) [7,8,9]. The *CCT* gene family can be classified into three sub-families, *COL* (CONSTANS-Like), *CMF* (CCT Motif Family) and *PRR* (Pseudo Response Regulator), according to their conserved domains [10]. COL proteins contain one or two BBOX domains and a CCT domain. CMF proteins contain only one CCT domain. PRR proteins share a response regulator (REC) domain, along with a CCT domain [11]. A phylogenetic analysis of CMF, COL and PRR proteins in Poaceae suggests that they evolved prior to the monocot/dicot divergence approximately 200 mya, and continuous *COL* genes’ evolution led to BBOX domains degradation, in which BBOX domains are reduced from two to one, and then to none [10].

Nowadays, *CCT* genes of the three sub-families are extensively studied in various plant species, including *Arabidopsis*, rice (*Oryza sativa*), maize (*Zea mays*) and wheat (*Triticum aestivum*) [7,11,12,13]. *CO* (*CONSTANS*), a *COL* sub-family gene, was the first cloned *CCT* gene controlling flowering time in *Arabidopsis* [7]. Under the long-day (LD) condition, *GI* (*GIGANTEA*) regulates circadian rhythms and *CO* expression; CO protein directly binds to the promoter of *FT* (*Flower Time*) to up-regulate its transcription. The latter moves from leaves to apical meristem, and induces a switch from vegetative to reproductive growth [9,14]. *Hd1*, the homolog of *CO* in rice, has a dual function in regulating flowering, as it activates *Hd3a* (*Heading date 3a*) expression, to promote heading under short day (SD) condition, but inhibits heading under the LD condition [15,16]. Another *COL* gene in rice, *DTH2*, promotes heading by inducing *Hd3a* and *RTF1* (*Rice Flowering Locus T1*) under LD condition [17]. Vernalization is indispensable for winter wheat. A *COL* gene in wheat, *VRN2* (*VERNALIZATION 1*) suppresses the expression of *VRN3* before vernalization, which activates the transcription of *VRN1*, the positive regulator of heading. Meanwhile, *VRN2* is suppressed by *VRN1* during vernalization, indicating that *VRN2* is a key switch for inflorescence development and heading [13,18]. Except for flowering time, the *COL* genes showed diverse functions in different plants. For instance, *COL4* is involved in salt stress response through the abscisic acid-dependent signaling pathway in *Arabidopsis* [19]. *OsGhd2* not only controls grain number, heading date and plant height, but also confers drought tolerance, by accelerating drought-induced premature senescence [20].

The second *CMF* sub-family genes exhibited multiplex bio-functions, which is similar to *COLs*. In maize, *ZmCCT* is correlated with photoperiod sensitivity and its allele from teosinte and tropical maize is consistently expressed at a higher level in temperate maize and confers later flowering under the LD condition [21]. Moreover, *ZmCCT* was reported to have influence on maize stalk rot resistance and tassel architecture, including tassel length, branch length and branch number [22,23]. *Ghd7* (*Grain number*, *plant height*, *and heading date 7*), a *CMF* gene in rice, represses *Edh1* (*Early heading date 1*) and *Hd3a* expression, thus delays heading under LD condition, but not SD condition [24]. Furthermore, *Ghd7* controls shoot branching by regulating *OsTB1* (*Teosinte Branched 1*) and *OsPHYB* (*PHYTOCHROME B*), and it also participates in multiple processes, including hormone metabolism and abiotic stress tolerance [25]. 

Differing from *COL* and *CMF* genes, the *PRR* sub-family genes are functionally conserved, in regulating photoperiodic flowering response through clock function. *AtPRR1*, also called *TOC1*, together with *CCA1* (*CIRCADIAN CLOCK ASSOCIATED 1*) and *LHY* (*LATE ELONGATED HYPOCOTYL*), comprise a feedback loop of circadian clock. In this feedback loop, CCA1 and LHY proteins inhibit *PRR1* expression around dawn, and PRR1 activate *CCA1* and *LHY* expression at night [26,27]. *OsPRR37* (also known as *DTH7* or *Ghd7.1*) suppresses the expression of downstream genes *Ehd1* and *Hd3a*, resulting in delayed flowering under the LD condition [28]. The deletions in the promoter of *TaPpd-H1* results in up-regulated expression, and alleles with these deletions are associated with photoperiod insensitivity and early heading [29,30].

Alfalfa (*Medicago sativa* L.) is regarded as the most important legume forage crop, which plays a vital role in livestock production [31]. An estimated 32 million ha of alfalfa were grown annually worldwide, and the global hay market in 2017 was 8.3 million metric tons (NAFA, 2016 and ITC, 2018). Floral transition is a key developmental switch that determines the production of dry matter in alfalfa; delayed flowering is a desirable trait to minimize damages from abiotic stresses and to enhance biomass yield via lengthening vegetative growth. On the other hand, genotypes with early flowering in spring are valuable to fill the seasonal forage gap, that exists due to winter dormancy [32,33]. Therefore, controlling flowering time is crucial for the reproductive success of alfalfa, and it has great impacts on regional adaptation, as well as biomass production. However, a systematic analysis on identification, classification, function and manipulation of genes controlling flowering time is still lacking in alfalfa. 

Compared with alfalfa that is an autotetraploid, allogamous and heterozygous species, *Medicago truncatula*, with the small and known genome information, is closely related to alfalfa. Genes from *M. truncatula* share high sequence identity to their counterparts from alfalfa [34,35]. Thus, it makes *M. truncatula* a model forage species for genetic studies. In the present study, we systematically analyzed the *CCT* gene family in *M. truncatula*, including their chromosomal distribution, gene structure, temporal and spatial expression profiles, response to rhythm, hormones and abiotic stresses. The results presented here would provide valuable information for the construction of a flowering regulatory network, as well as utilization of the *CCT* gene family, in both *M. truncatula* and alfalfa. 

## 2. Results

### 2.1. Identification of CCT Genes in M. truncatula Genome

We used the HMM file corresponding to the CCT domain (PF06203) as a query to search the *M*. *truncatula* protein database, and retrieved a total of 51 putative CCT protein sequences with default parameters. The existence of the conserved CCT domain was confirmed by SMART, and redundant sequences were removed. Finally, 36 MtCCT protein sequences were identified and the corresponding genes were designated as *MtCCT1* to *MtCCT36*, based on their chromosome locations retrieved from the Ensemble Plants Database (Table 1). Sequence analyses revealed that these MtCCT proteins varied greatly in length, from 121 to 796 aa. The protein molecular weight of these MtCCT proteins ranged from 13.59 KD (MtCCT17) to 87.25 KD (MtCCT18), and the values of their isoelectric points ranged from 4.47 (MtCCT5 and MtCCT14) to 9.86 (MtCCT17) (Table 1).

### 2.2. Analyses of Gene Structure, Conserved Domain and Phylogenetic Relationship of MtCCT Genes 

**Figure 1:**
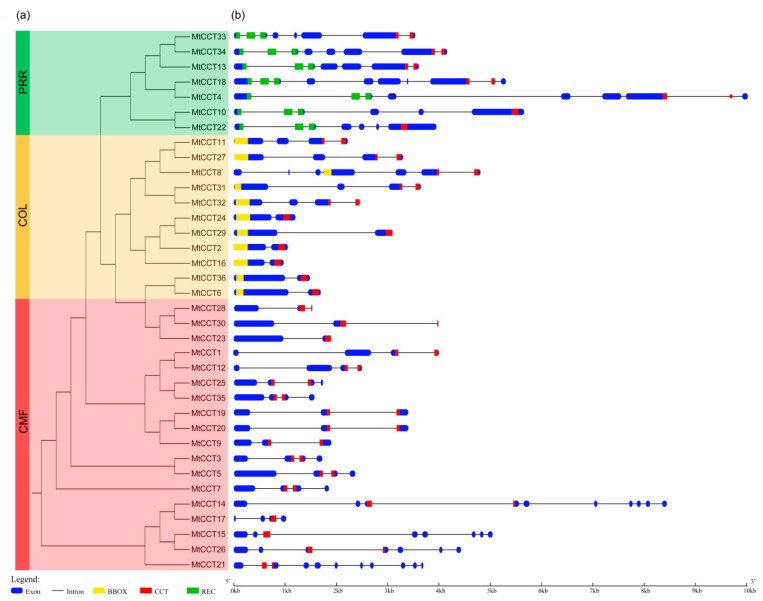
Phylogenetic relationship, gene structure and architecture of conserved domains of *MtCCT* genes. (**a**) Phylogenetic tree of *MtCCT* genes (36 in total) was constructed based on the full-length sequences of proteins using MegAlign. Different subfamilies are highlighted in different colors: *PRR* in green, *COL* in yellow, and *CMF* in red. (**b**) Exon-intron structure and conserved domains of *MtCCT* genes. Blue boxes indicate exons; black lines indicate introns; red, yellow and green boxes indicate CCT (CO, COL and TOC1), BBOX (B-box-type zinc finger) and REC (cheY-homologous receiver) domains, respectively.

The corresponding CDS and genomic sequences of 36 *MtCCT* genes were analyzed by using the GSDA software. The exon and intron structures of the 36 *MtCCT* genes were shown in Figure 1, illustrating that *MtCCT* genes varied considerably in sequence length and structure. Concretely, *MtCCT17* gene has the shortest sequence with a length of 1019 bp, and *MtCCT1* gene has the longest sequence, with a length of 10,019 bp. In particular, seven genes (*MtCCT2*, *MtCCT6*, *MtCCT16*, *MtCCT23*, *MtCCT24, MtCCT29* and *MtCCT36*) showed a similar and relatively simple gene structure with only one intron, while *MtCCT21* has 10 introns. The remaining *MtCCT* genes contain 2 to 9 introns (Figure 1).

Multiple sequence alignment showed that all of the 36 MtCCT proteins contain a conserved CCT domain, 11 MtCCT proteins contain a conserved BBOX domain, and 7 MtCCT proteins contain a conserved REC domain (Figure 1 and Figure S1). The motifs of these three conserved domains were identified by using MEME software. It also demonstrated that CCT domain was more conserved than BBOX domain and REC domain in *M*. *truncatula* (Figure S2).

Based on the multiple sequence alignment of MtCCT proteins, we further preformed the phylogenetic analysis of *MtCCT* genes. The results showed that *MtCCT* genes could be grouped into three distinct sub-clusters. The first sub-cluster included 7 *MtCCT* genes, which belong to the *PRR* subfamily, and they all contain both CCT and REC domains. The second sub-cluster included 11 *MtCCT* genes, which belong to the *COL* subfamily, and they contain both CCT and BBOX domains. The third sub-cluster included 18 *MtCCT* genes, which belong to the *CMF* subfamily, and they contain only the CCT domain (Figure 1). It was obvious that the phylogenetic clustering of the *MtCCT* gene is consistent with the distribution of their conserved domain.

### 2.3. Analyses of Chromosomal Distribution and Synteny of MtCCT Genes

A map of the chromosomal distributions of *MtCCT* genes constructed based on their physical position information illustrated that they were unevenly distributed on seven chromosomes (Chr1, 2, 3, 4, 5, 7 and 8) (Figure S3). Among them, eight *MtCCT* genes localize on Chr 4 (22.2%), seven on Chr 3 (19.4%), six on Chr 1 (16.7%), five on Chr 7 (13.8%), four on Chr 5 (11.1%), three on Chr 2 (8.3%), and three on Chr 8 (8.3%). Additionally, the analysis on the duplication events of the *MtCCT* genes showed that one *MtCCT* gene pair (*MtCCT19/20*) located on Chr 4 could be identified as tandem duplication. Interestingly, the sequences of *MtCCT19* and *MtCCT20* were completely identical.

**Figure 2:**
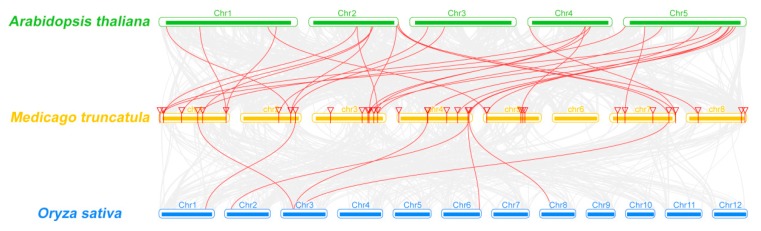
Synteny analyses of *MtCCT* genes between *M. truncatula* and *A. thaliana*/*O. sativa*. Gray lines in the background indicate the collinear blocks within *M. truncatula*, and *A. thaliana*/*O. sativa*, and the red lines highlight the syntenic *MtCCT* gene pairs.

Moreover, we carried out comparative syntenic analysis on *MtCCT* genes with another two representative species, *A. thaliana* (dicot) and *O. sativa* (monocot) (Figure 2). A total of 22 *MtCCT* genes showed syntenic relationship with those in *Arabidopsis*, and 28 corresponding orthologs were identified in *A. thaliana*. Meanwhile, only seven *MtCCT* genes showed syntenic relationships with those in *O. sativa*, and seven corresponding orthologs were identified in *O. sativa* (Table S1). Among these orthologous pairs, six *MtCCT* genes (*MtCCT5*, *MtCCT19/20*, *MtCCT23*, *MtCCT24* and *MtCCT32*) had their corresponding orthologs both in *A. thaliana* and *O. sativa*, suggesting their potentially critical roles in plant growth and development.

### 2.4. Phylogenetic Analysis of CCT Proteins in Plants

**Figure 3:**
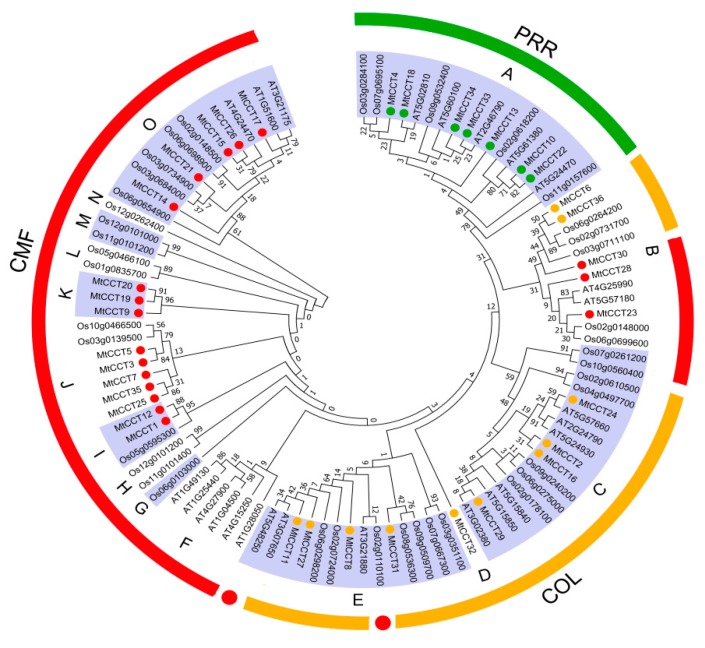
Phylogenetic relationships among *CCT* genes in *M. truncatula*, *Arabidopsis* and rice. The phylogenetic tree was constructed using MEGA (Molecular Evolutionary Genetics Analysis) 7 based on (ML, Maximum Likelihood) method; bootstrap was 1000 replicates. The different colored circles indicate proteins of different subfamilies.

To evaluate the evolutionary relationship of the CCT proteins from representative plant species, we conducted a phylogenetic three based on 101 full-length CCT protein sequences, including 36 from *M*. *truncatula*, 25 from *Arabidopsis*, and 40 from rice (Figure 3). The results showed that 101 CCT proteins were clustered into three sub-clusters with 15 groups (from A to O). Among them, group A contained 17 CCT proteins from all three species, and it belonged to the PRR sub-family. Another 10 groups (F, G, H, I, J, K, L, M, N and O) contained 40 CCT proteins from all three species, and they belonged to the CMF subfamily. Three groups (C, D and E) containing 32 CCT proteins from three species belonged to the COL subfamily, except one AtCCT (AT5G48250) and three OsCCTs (Os02g0110100, Os02g0610500 and Os04g0497700). Interestingly, group B, consisting of 12 CCT proteins from three species, belonged to the COL and CMF subfamilies, respectively. The former contained five CCT proteins from *M*. *truncatula* and rice, and the latter included seven CCT proteins from all three species (Figure 3). These results indicated that CCT proteins occurred before the divergence of monocot/dicot, and that orthologous proteins might have diversified afterwards among plant species. Moreover, group B, C and E, containing both COL and CMF subfamily members, suggested that CMF subfamily members might be evolved from the COL subfamily, due to the degradation of one or two BBOX domains. 

### 2.5. Temporal and Spatial Expression Profiling of MtCCT Genes 

**Figure 4:**
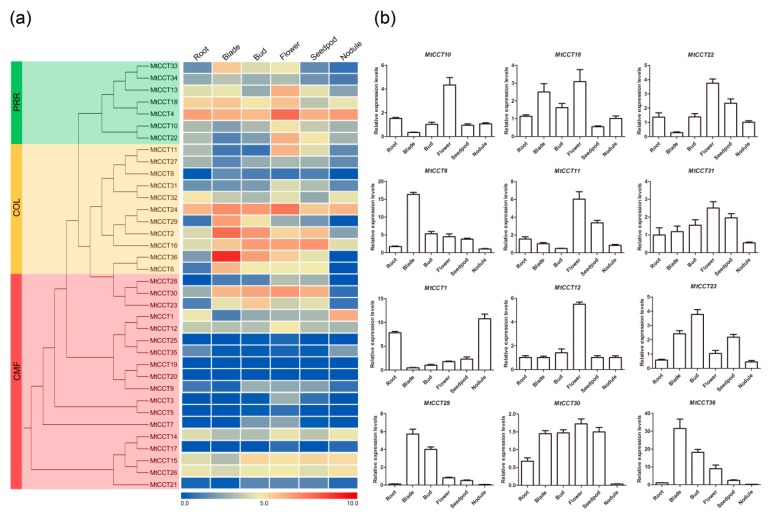
Expression files of *MtCCT* genes in six representative tissues (Root, Blade, Bud, Flower and Seedpod). (**a**) The expression heatmap of *MtCCT* genes constructed by electronic expression data; (**b**) The expression patterns of 12 *MtCCT* genes in six tissues were examined by qPCR(quantitative PCR). Expression levels are normalized to *MtACTIN* and error bars indicate standard deviation among three biological replicates.

Gene expression patterns provide important clues on gene function. To illustrate the role of *MtCCT* genes during *M*. *truncatula* growth and development, the electronic expression data in six tissues (i.e., root, blade, bud, flower, seedpod and nodule) were retrieved from a public available RNA-sequencing database (Table S2). Amazing HeatMap software was used to generate the heatmap. The results showed that the *MtCCT* genes had various transcript levels in six tissues. Among them, *MtCCT4*, *MtCCT14*, *MtCCT15*, *MtCCT24* and *MtCCT26* were highly and consistently expressed in six tissues; whereas, *MtCCT5*, *MtCCT8*, *MtCCT17*, *MtCCT21*, *MtCCT25* and *MtCCT35* were expressed at relatively low level in six tissues (Figure 4a). On the other hand, several genes were highly expressed in specific tissues, for instance, *MtCCT1* in root and nodule, *MtCCT10*, *MtCCT11*, *MtCCT12*, and *MtCCT31* in flower, *MtCCT29* in blade and bud, and *MtCCT22* in flower and seedpod. In addition, other genes were expressed at low level in specific tissues, for instance, *MtCCT6*, *MtCCT23*, *MtCCT30* and *MtCCT36* in root and nodule, and *MtCCT18* in seedpod (Figure 4a).

The expression levels of the above-mentioned twelve *MtCCT* genes were further confirmed through qRT-PCR in the same tissues (Figure 4b). It showed that the expression level of these twelve *MtCCT* genes were consistent with those in Figure 4a,b. These data together indicated that these twelve *MtCCT* genes participate in the growth and development in specific tissues in *M*. *truncatula*, functioning alone or in combination.

Additionally, we also analyzed the expression levels of 16 *MtCCT* genes with available microarray data, during seed and nodule development in *M*. *truncatula* (Table S2). The expression levels of *MtCCT6*, *MtCCT12* and *MtCCT36* gradually decreased with seed maturity, suggesting that these genes played critical roles in cell division or proliferation in seed development. Meanwhile, the expression levels of *MtCCT32*, *MtCCT33* and *MtCCT11* gradually increased with seed maturity, suggesting they may be involved in the accumulation of dry matter during seed development (Figure S4). During nodule formation, the expression levels of three *MtCCT* genes (*MtCCT26*, *MtCCT1* and *MtCCT15*) were up-regulated, whereas one (*MtCCT16*) was down-regulated. It also showed that the expression levels of five genes (*MtCCT11*, *MtCCT13*, *MtCCT22*, *MtCCT32* and *MtCCT33*) increased at the early nodulation stage, and then decreased sharply during the later nodulation stage (Figure S4). These results together indicated that they might be involved in nitrogen fixation during the nodule development in *M*. *truncatula*.

### 2.6. Expression Patterns of MtCCT Genes under Light Circle

**Figure 5:**
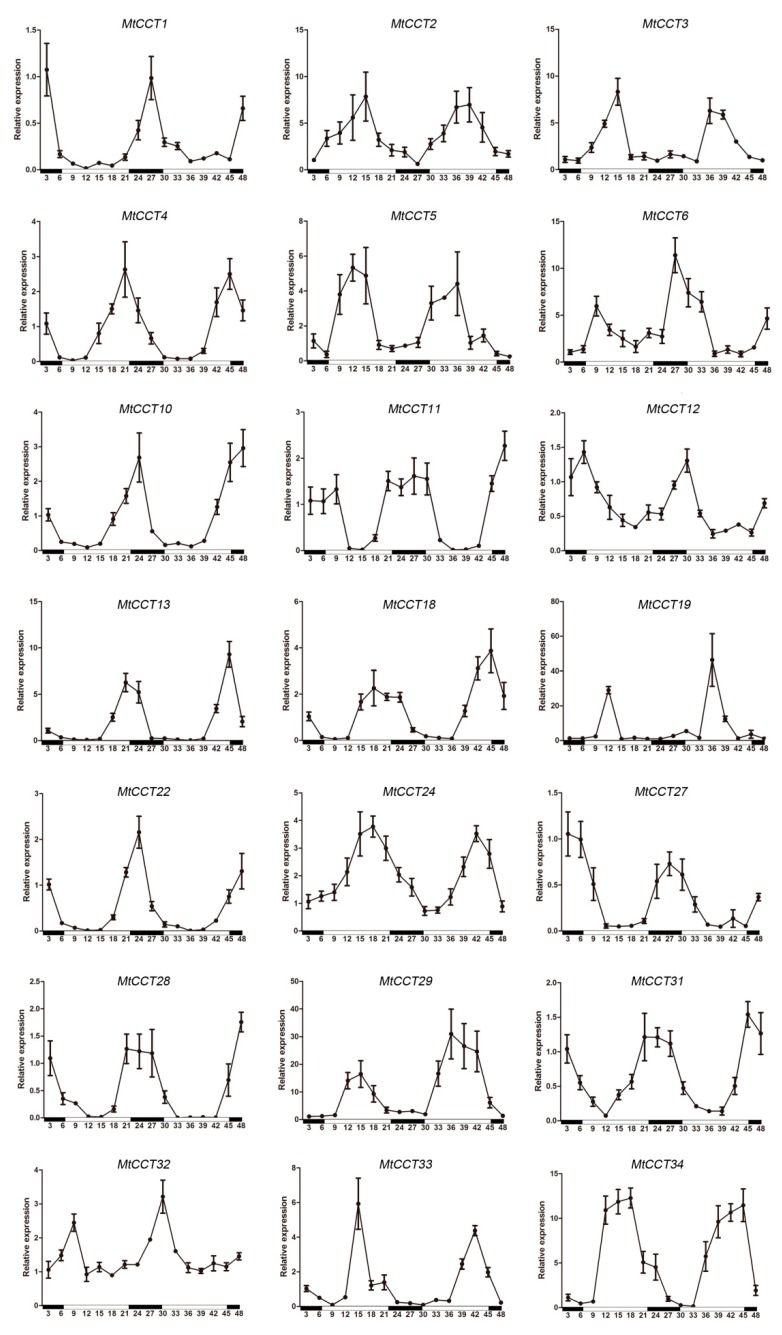
Diurnal expression patterns of *MtCCT* genes’ response to light circle. The white and black bars represent the light and dark periods, respectively. Expression levels are normalized to *MtACTIN* and error bars indicate standard deviation among three biological replicates.

It has become clear that plants are richly rhythmic, and many aspects of plant biology, including photoperiodic flower induction, petal movement, floral fragrance emission and resistance to abiotic stress, exhibit circadian rhythmicity [36]. We thus investigated the expression patterns of *MtCCT* genes under light circle. It showed that 22 out of 36 *MtCCT* genes (i.e., *MtCCT1*, *MtCCT2*, *MtCCT3*, *MtCCT4*, *MtCCT5*, *MtCCT6*, *MtCCT10*, *MtCCT11*, *MtCCT12*, *MtCCT13*, *MtCCT18*, *MtCCT19/20*, *MtCCT22*, *MtCCT24*, *MtCCT27*, *MtCCT28*, *MtCCT29*, *MtCCT31*, *MtCCT32*, *MtCCT33*, and *MtCCT34*) displayed an obvious circadian rhythm over 24 h, suggesting that these genes could respond to light changes, during their regulation on plant growth and development (Figure 5). Consistent with their circadian expression pattern, 18 of these genes contained 1-4 light responsive *cis*-element (YTCANTYY) in their promoter regions, except *MtCCT24*, *MtCCT27* and *MtCCT29* (Figure S5). 

Among the 22 *MtCCT* genes’ response to circadian rhythm, 10 of them (*MtCCT2*, *MtCCT3*, *MtCCT5*, *MtCCT6*, *MtCCT19/20*, *MtCCT24*, *MtCCT29*, *MtCCT33*, and *MtCCT34*) were mainly expressed during daytime and inhibited in dark, and their expression levels peaked at midday (Figure 5). Meanwhile, seven of them (*MtCCT1*, *MtCCT10*, *MtCCT11*, *MtCCT22*, *MtCCT27*, *MtCCT28* and *MtCCT31*) were mainly expressed in dark and inhibited by light, and their expression levels peaked at midnight (Figure 5). Moreover, *MtCCT4*, *MtCCT13* and *MtCCT32* expressed according to the light and peaked around evening, while *MtCCT12* and *MtCCT18* expressed according to the dark and peaked around dawn. These results demonstrated that these *MtCCT* genes with various circadian rhythm patterns participate in multiple bio-functions through different pathways in *M*. *truncatula*.

### 2.7. Expression Patterns of MtCCT Genes in Response to Hormones

Previous studies have demonstrated that *CCT* genes are key regulators of flowering time in cereals (Li and Xu, 2017). Temporal and spatial expression profiles suggested that *MtCCT* genes function in plant growth and development, including seed and nodule development (Figure 4 and Figure S4). We therefore inferred that *MtCCT* genes regulate these bio-processes through plant hormones. Meanwhile, several *cis*-elements related to phytohormones were identified within the promoter regions of *MtCCT* genes, including ABA response element (ABRE) motif, auxin response element (AuxRE) motif, auxin response factors (ARF) motif, ethylene response element (ERE) motif, gibberellin acid response element (GARE and TATC) motifs (Figure S5). 

**Figure 6:**
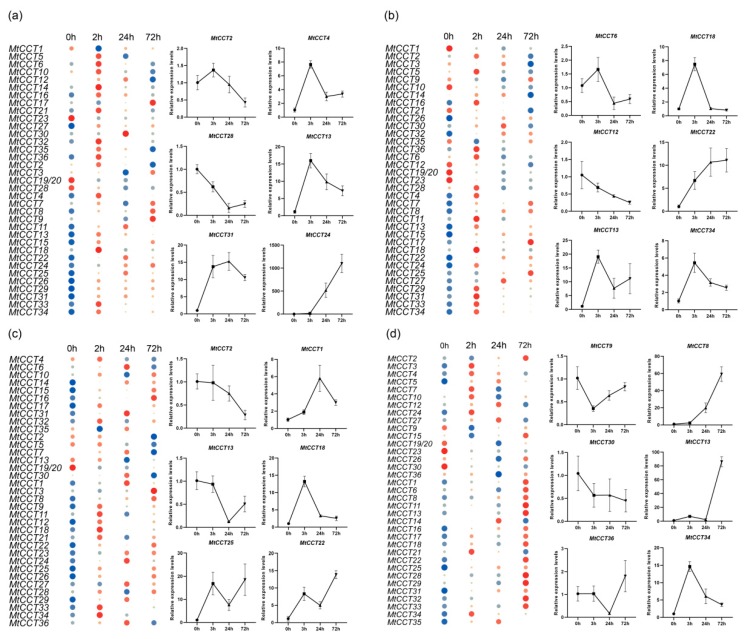
Expression of *MtCCT* genes in response to different hormones. Plants with fourth blade were treated with 1 mM IAA (**a**), 1 mM GA_4_ (**b**), 1 mM SA (**c**) and 1 mM ABA (**d**). Expression levels are normalized to *MtACTIN* and error bars indicate standard deviation among three biological replicates. The relative expressions levels are -log2 transformed and visualized for heat map. The colors vary from blue to red, and circles from small to large represent the scale of the relative expression levels.

We further investigated the expression profiles of *MtCCT* genes in response to different hormone treatments by using qRT-PCR (Table S2). It showed that 15, 14, 10 and 9 *MtCCT* genes were not responsive to IAA, GA, SA or ABA treatment, but the expression levels of other genes distinctly responded to these hormones, with two fold as threshold (Figure 6). In total, 16 and 5 *MtCCT* genes were respectively up-regulated and down-regulated under IAA treatment (Figure 6a), 16 and 6 under GA treatment (Figure 6b), 19 and 7 under SA treatment (Figure 6c), and 19 and 8 under ABA treatment (Figure 6d). It was obvious that *MtCCT* genes responded differently to specific hormones. 

Additionally, the up-regulated and down-regulated *MtCCT* genes’ response to hormones showed distinct expression patterns, and the typical *MtCCT* genes were selected for further illustration (Figure 6). For instance, the expression levels of *MtCCT24*, *MtCCT22* and *MtCCT8* were persistently increased under the treatment of IAA, GA and ABA, respectively. Meanwhile, the expression levels of *MtCCT24* and *MtCCT30* were persistently decreased in response to GA and ABA. The peak expression levels of *MtCCT* genes were different in response to four hormones; *MtCCT13*, *MtCCT34* and *MtCCT18* peaked at 3 h in response to IAA, GA and SA; *MtCCT31* and *MtCCT1* peaked at 24 h in response to IAA and SA; *MtCCT4*, *MtCCT13* and *MtCCT22* showed oscillating expression patterns in response to IAA, GA and SA, as well as *MtCCT* 13 in response to ABA (Figure 6). 

Notably, several genes were dramatically up-regulated under different hormone treatments (Figure 6). The expression level of *MtCCT24* increased more than 1000 times under the treatment of IAA; the expression level of *MtCCT25* was induced by 30 times in response to GA; likewise, the expression levels of *MtCCT13* and *MtCCT31* increased more than 30 times under the treatment of ABA (Table S2). Our results provided a clear guide for the functional characterization of these *MtCCT* genes.

### 2.8. Expression of MtCCT Genes Which Responded to Abiotic Stresses 

**Figure 7:**
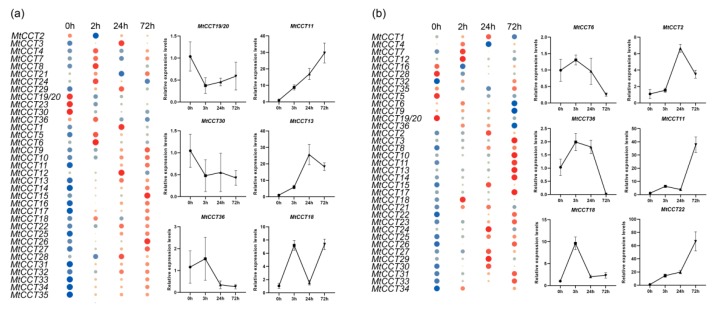
Expression of *MtCCT* genes in response to abiotic stress. Plants with fourth blade were watered with 0.3 M NaCl (**a**) and 17% PEG (polyethylene glycol) (**b**). Expression levels are normalized to *MtACTIN* and error bars indicate standard deviation among three biological replicates. The relative expressions levels are -log2 transformed and visualized for heat map. The colors vary from blue to red, and circles from small to large represent the scale of the relative expression levels.

Abiotic stress always induces gene expression to protect plant cell from injury. To decipher the roles of *MtCCT* genes in response to abiotic stress, we analyzed the expression profiles of 36 *MtCCT* genes upon NaCl and PEG treatment, to simulate salinity and acute dehydration condition. The results showed that 23 and 22 *MtCCT* genes were up-regulated by NaCl or PEG treatment, respectively, and, 4 and 5 *MtCCT* genes were down-regulated (Figure 7, Table S2). The up-regulated and down-regulated *MtCCT* genes responded to abiotic stress with distinct expression patterns, suggesting their variable sensitivity to NaCl and PEG treatment (Figure 7). In brief, *MtCCT11* and *MtCCT22* expression continually increased in response to NaCl and PEG, respectively. The expression of *MtCCT18* peaked at 3 h simultaneously under the treatment of NaCl and PEG; whereas, *MtCCT13* and *MtCCT22* peaked at 24 h. *MtCCT30* and *MtCCT11* showed an oscillating expression pattern in response to NaCl and PEG (Figure 7a,b). Additionally, the expression levels of *MtCCT25*, *MtCCT31* and *MtCCT33* dramatically increased by more than 25 times under NaCl treatment (Figure 7a), and the expression levels of *MtCCT11* and *MtCCT22* were induced 30 times under PEG treatment (Figure 7b).

**Figure 8:**
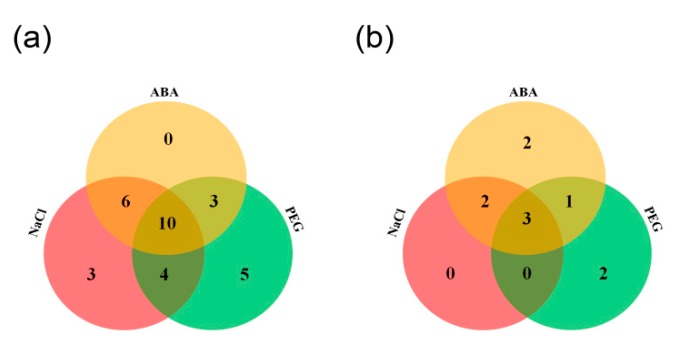
The detailed numbers of simultaneously up-regulated (**a**) and down-regulated (**b**) *MtCCT* genes by ABA (abscisic acid), NaCl and PEG.

Abscisic acid (ABA) plays critical roles in plant abiotic stress tolerance; the expression of *MtCCT* genes induced by salinity or dehydration could also be induced by ABA. Therefore, we also investigated the expression levels of salt and dehydration-responsive genes under ABA treatment. It showed that 10 genes (*MtCCT11*, *MtCCT13*, *MtCCT14*, *MtCCT17*, *MtCCT18*, *MtCCT22*, *MtCCT25*, *MtCCT31*, *MtCCT33* and *MtCCT34*) were simultaneously up-regulated under NaCl, PEG and ABA treatments, while three genes (*MtCCT19/20* and *MtCCT36*) were simultaneously down-regulated under three treatments (Figure 8). Moreover, six (*MtCCT1*, *MtCCT6*, *MtCCT16*, *MtCCT28*, *MtCCT32* and *MtCCT35*), four (*MtCCT15*, *MtCCT16*, *MtCCT26* and *MtCCT27*) and three (*MtCCT8*, *MtCCT21* and *MtCCT29*) genes could be induced by the combinations of NaCl/ABA, NaCl/PEG, PEG/ABA, respectively (Figure 8a), whereas, two (*MtCCT23* and *MtCCT30*) and one (*MtCCT9*) genes were down-regulated by the combinations of NaCl/ABA, PEG/ABA, respectively (Figure 8b). Among the genes that were up-regulated, three (*MtCCT5*, *MtCCT9* and *MtCCT12*) and five (*MtCCT2*, *MtCCT3*, *MtCCT23*, *MtCCT24* and *MtCCT30*) genes were only induced by NaCl and PEG, respectively; while two (*MtCCT5* and *MtCCT9*) and two (*MtCCT15* and *MtCCT26*) genes were only down-regulated by PEG and ABA (Figure 8, Table S2). This result suggests that these *MtCCT* genes might play vital roles in the protection of plants from abiotic injury, and they may participate in the regulation of abiotic stresses in the ABA-dependent or ABA-independent manner in *M*. *truncatula*.

## 3. Discussion

Flowering time is one of the most important agronomic traits for crops. Optimal flowering time is indispensable to maximize crop yield and adaptation in production. Early flowering may be a desirable trait for cereal crops whose seeds are the harvested product, but late flowering could be advantageous when total biomass is the objective production, as is the case for forage crops. It is important to elucidate flowering time related genes and their regulation mechanism in alfalfa and other legumes. The CCT domain-containing genes (*CCT* genes) are the major genetic determinants that regulate the flowering time in plants [5]. The completion of the *M*. *truncatula* genome sequencing made it possible to investigate *CCT* genes and their potential functions involved in flowering time, which could be applied in alfalfa molecular breeding. 

In the present study, a total of 36 *MtCCT* genes distributed across seven chromosomes were identified in *M*. *truncatula* (Table 1). An analysis on their conserved motifs revealed that they could be classified into three subfamilies, with seven *MtCCT* genes belonging to the *PRR* subfamily, and 11 *MtCCT* genes to the *COL* subfamily (Figure 1). An analysis on gene structure suggested that *MtCCT* genes varied considerably in sequence length and structure (Figure 1). A phylogenetic analysis of all CCT proteins from *M. truncatula*, *Arabidopsis* and rice showed that they were clustered into three subfamilies or 15 distinct groups (Figure 3), indicating *CCT* genes occurred before the differentiation of monocot and dicot. Additionally, members of the CMF subfamily were not as conserved as members of the PRR or COL subfamilies, suggesting that members of the CMF subfamily might function in a species-specific manner. Nevertheless, members of the PRR and COL subfamily might have similar biological functions in plants. For instance, *CO* (AT5G15840) and *Hd1* (Os6g0275000) in group C, belonging to the COL subfamily, are involved in the regulation of flowering time in *Arabidopsis* and rice, respectively [7,15]. Thus, their corresponding homologs *MtCCT29* may have a similar function and be associated with flowering time in *M*. *truncatula*. However, *CO* promoted flowering under LD condition in *Arabidopsis*, whereas *Hd1* has a dual function in regulating flowering, which promotes flowering under SD condition, but inhibiting the heading under LD condition [7,15]. The regulation mechanism in flowering time differed greatly between *Hd1* in rice and *CO* in *Arabidopsis*, suggesting that the specific function of different members in the same group varied greatly among plant species. Therefore, apart from referencing the orthologous genes, the identification and characterization of the individual *MtCCT* gene are also essential for the in-depth investigation of plant functional genomics. 

In this study, we found that chromosome 4 contained the most *MtCCT* genes, with eight members on this chromosome. Interestingly, the sequences of *MtCCT19* and *MtCCT20* were completely identical, and *MtCCT18* was present at 200 kbp upstream *MtCCT19* and *MtCCT20* on chromosome 4 (Figure S3). These three genes formed a gene cluster, and this cluster may be involved in regulating the flowering time in *M. truncatula*. In many plant species, a gene cluster was often found in modulating an important trait. For an example, a gene cluster of three genes encoding lectin receptor kinases confers brown planthopper resistance in rice [37]. In another study, five genes *AtGRXS3/4/5/7/8* were arranged in a tandem array, as a gene cluster on *Arabidopsis* chromosome 4, and these five genes shared virtually identical regulatory patterns, collectively acting to modulate primary root growth in response to soil nitrate [38]. Therefore, the gene cluster of *MtCCT18*, *MtCCT19* and *MtCCT20* are potential key players in the flowering time in *M. truncatula*, which is worth further investigation.

Genes specifically expressed in certain organs are critical for corresponding organogenesis in *M. truncatula*. For instance, *MtSWEET11*, a nodule-specific sucrose transporter isolated from the *M. truncatula* root nodule, has been reported to be involved in sucrose distribution within the nodule [39]. A demethylase gene, *DEMETER* (*DME*), is spatially regulated within the nodule of *M. truncatula*. Another previous report showed that *DME* in *M. truncatula* is essential for nodule development and it regulates the expression of 1425 genes, thereby leading to the transcriptional activation of nodule differentiation genes [40]. In this study, we identified genes that displayed highly transcript levels in specific tissues, e.g., *MtCCT1* in root and nodule, *MtCCT10*, *MtCCT11*, *MtCCT12*, and *MtCCT31* in flower, *MtCCT29* in blade and bud, and *MtCCT22* in flower and seedpod. Meanwhile, some genes were expressed at low level in specific tissues, e.g., *MtCCT6*, *MtCCT23*, *MtCCT30* and *MtCCT36* in root and nodule, and *MtCCT18* in seedpod (Figure 4). These expression profiles will contribute to the functional clarification of the above *MtCCT* genes.

It was reported that *CCT* genes regulate flowering in two main pathways: circadian clock-controlled flowering and photoperiod-regulated flowering [5,12]. Studies on the gene expression of *CCT* genes in the circadian clock pathway in *Arabidopsis* are relatively more advanced. The *PRR5*, *PRR7* and *PRR9* of the *CCT* genes in *Arabidopsis* are expressed at different time points of the day and they are collectively essential to proper timekeeping [41]. Furthermore, the expressions of several *CCT* genes in *Aegilops tauschii* showed obvious circadian rhythmic expression patterns within 24 h [42]. Additionally, *ZmCOL3* is a *CCT* gene that represses flowering in maize by interfering with the circadian clock and activating the expression of another *CCT* genes *ZmCCT* [12]. In our study, we found that the expression of 22 *MtCCT* genes is responsive to circadian rhythm, and most of these genes contained 1-4 light responsive *cis*-elements in their promoter regions (Figure 5 and Figure S5). However, the circadian rhythm patterns of these *MtCCT* genes varied, and the detailed regulatory mechanism of each *MtCCT* gene in flowering and other bio-functions should be further studied.

The growth and development processes of plants are closely associated with phytohormones. The phytohormone GA is known to play an important role in regulating the timing of floral transition. GA induces early flowering under short-day light conditions by regulating the floral meristem-identity gene *LEAFY* and the flowering-time gene *SUPPRESSOR OF OVEREXPRESSION OF CO 1* (*SOC1*) [43,44]. IAA may be involved in the regulating of flowering time under SD conditions in a GA-dependent manner, but the exact mechanism remains unclear [45]. Furthermore, ABA affects flowering through two independent regulatory networks: the activation of GI and CO functions upstream of the florigen and the down-regulation of *SOC1* signaling [46]. The expression profiling of *MtCCT* genes in response to hormones demonstrated that most *MtCCT* genes were regulated by multiple hormones with distinct sensitivity (Figure 6). Notably, several genes (*MtCCT24*, *MtCCT25*, *MtCCT13* and *MtCCT31*) were dramatically up-regulated under hormone treatment. These results suggest that the function of *MtCCT* genes have considerably differentiated and the hormone regulatory pathways involved have diversified. It will be worth further investigation on how *MtCCT* genes are regulated by different hormones in plant growth and development.

Recent studies have shown that *CCT* genes have multiple functions in plant abiotic stress tolerance. *OsGhd2* (*Os02g0731700*) confers drought tolerance by accelerating drought-induced premature senescence, and controls grain number, heading date and plant height [20]. Its homologs in *M. truncatula*, *MtCCT6* and *MtCCT36* showed a significantly low expression level under PEG treatment (Table S2). Moreover, the expression levels of several genes (*MtCCT25*, *MtCCT11*, *MtCCT22*, *MtCCT31* and *MtCCT33*) were dramatically regulated by salinity and dehydration stresses, suggesting their critical roles in the abiotic stress tolerance in *M*. *truncatula* (Figure 7). Development and stress tolerance are dynamically balanced during plant growth, and ABA plays a key role in the crosstalk between abiotic stress and growth [22]. *COL4 (AT5G57660)* is involved in the salt stress response, through the ABA-dependent signaling pathway in *Arabidopsis* [19]. However, its homolog, *MtCCT24*, was dramatically induced by PEG, but not by NaCl (Figure 8). On the other hand, 10 and 3 *MtCCT* genes were up-regulated and down-regulated simultaneously by NaCl, PEG and ABA, respectively, suggesting that they might participate in abiotic stresses’ regulatory network in a ABA-dependent manner. The strong response of *MtCCT* genes to abiotic stresses demonstrated that a set of *MtCCT* genes play critical roles in the prevention of plant against abiotic injury in *M*. *truncatula*.

Collectively, our study suggested that *MtCCT* genes play critical roles, not only in regulating flowering time, but also in plant growth and development, as well as in abiotic stress tolerance. Considering that the detailed regulatory mechanisms underlining *CCT* genes are still not clear in *M. truncatula*, it will be of great interest to elucidate individual *MtCCT* genes in the neat further. For instance, further characterization of *MtCCT29* would provide a great advantage for alfalfa breeding with ideal flowering time; *MtCCT13* could be a key player in nodule development in *M. truncatula*; and *MtCCT25*, *MtCCT11*, *MtCCT22*, *MtCCT31* and *MtCCT33* might contribute to abiotic stress tolerance in *M. truncatula*. Nevertheless, our current systematic studies provided a gene expression atlas for *CCT* genes in *M. truncatula*, which could be a valuable reference in its close relative alfalfa in functional genomics studies, as well as in molecular breeding. 

## 4. Materials and Methods 

### 4.1. Plant Materials

*M*. *truncatula* (cv. Jemalong A17) seeds were sterilized in 75% ethanol for 5 min, rinsed with sterile water 5 times, and then placed on moistened filter paper in dishes. They were subsequently cultured in a growth chamber at 25 °C. Seven-day-old seedlings were then subjected to different experiments. For each experiment, all samples were immediately frozen in liquid nitrogen and stored at −80 °C for RNA extraction.

For the temporal and spatial expression analyses of *CCT* genes, the seedlings were transferred into pots (20 cm in diameter) with nutritional soil and grown in a greenhouse under 16/8 h light/dark regime at 25 °C. Roots, blades, buds, flowerings, seedpods and nodules were collected at flowering stage from three individual plants with similar growth conditions. 

For the expression analyses of *CCT* genes’ response to light, the seedlings were transferred into 10 × 10 cm pots filled with nutritional soil and grown in an artificial climate room under a 16/8 h light/dark regime at 25 °C. The latest expanded blades were collected every 3 h for a continuous 48h from three individual plants at squaring stage. 

For the expression analyses of *CCT* genes’ response to different treatments, the 7-day-old seedlings were transferred into flask with 1/2 MS liquid medium and grown in a controlled growth cabinet under 16/8 h light/dark regime at 25 °C. Ten days later, the plants with the fourth leaf expanded were watered with 1 mmol·L^−1^ IAA, 1 mmol·L^−1^ GA4, 1 mmol·L^−1^ SA, 1 mmol·L^−1^ ABA, 15% PEG6000 and 300 mmol·L^−1^ NaCl, respectively. For each treatment, the whole plant was collected at 0 h, 2 h, 24 h and 72 h with a triplicate.

### 4.2. Identification of CCT Genes in M. truncatula

The genome and protein sequences of *M. truncatula* were downloaded from the EnsemblePlants Database (http://plants.ensembl.org/index.html). The hidden Markov model (HMM) file of the CCT family (PF06203) was retrieved from the Pfam Database (https://pfam.sanger.ac.uk/). The CCT protein sequences of *M. truncatula* were aligned using the HMM model of the HMMER3.0 (http://hmmer.org/), with an *E*-value of 0.001 and the removal of redundant sequences. The non-redundant sequences were submitted to the SMART database (http://smart.embl-heidelberg.de/) to verify the existence of the conserved CCT domain. The tools from the ExPASy website (https://web.expasy.org/protparam/) were used to analyze the sequence length, molecular weight and isoelectric point of the identified MtCCT proteins. The corresponding *MtCCT* genes were obtained from the genome database of *M. truncatula*.

### 4.3. Analyses of Gene Structure and Conserved Motif

The exon-intron structures of *MtCCT* genes were drawn with the online Gene Structure Display Server (GSDS 2.0; http://gsds.cbi.pku.edu.cn), based on the CDS and corresponding full-length sequence. The conserved motifs in MtCCT proteins were identified using the MEME software (http://meme-suite.org/tools/meme). The parameters were set as follows: width of each motif was 10-100 amino acid residues, maximum number of motifs was 3, and other parameters of default values.

### 4.4. Analyses of Chromosomal Distribution, Synteny and Cis-Elements of MtCCT Genes

Based on the location data of the *MtCCT* genes and chromosomal lengths of *M*. *truncatula*, we drew the schematic diagram of chromosomal distributions of *MtCCT* genes by using MG2C software (http://mg2c.iask.in/mg2c_v2.0/). The syntenic relationship between *MtCCT* genes and *CCT* genes from *Arabidopsis* and rice was determined by using Dual Synteny Plotter software (http://chibba.pgml.uga.edu/mcscan2/).

The 1500-bp promoter sequences upstream to the start code (ATG) of *MtCCT* genes were extracted from *M*. *truncatula* genome sequences, and the regulatory *cis*-elements in the promoter of *MtCCT* genes were predicted by the PlantCARE Database (http://bioinformatics.psb.ugent.be/webtools/plantcare/html/).

### 4.5. Phylogenetic Analysis of CCT Proteins in Representative Plants

A total of 101 CCT proteins, including 36 from *M*. *truncatula*, 25 from *Arabidopsis*, and 40 from rice, that were downloaded from the UniProt database (https://www.uniprot.org), were used for the phylogenetic analysis of CCT proteins in plants. The 101 CCT protein sequences were aligned with ClustalX software (https://clustalx.software.informer.com/2.1/) with default parameters, and the phylogenetic tree was then constructed with MEGA7.0 software (https://mega.software.informer.com/7.2/), using the Maximum Likelihood (parameter setting: Bootstrap method (1000 replicates), Poisson model, pairwise deletion). The phylogenetic tree was constructed based on the multiple sequence alignment in MegAlign (https://www.dnastar.com/software/lasergene/), by using the Clustal W method.

### 4.6. RNA Extraction and Real-Time Quantitative PCR

Total RNAs were extracted with Eastep^TM^ Super Total RNA Extraction Kit (Promega, LS1040, Beijing, China) following the manufacturer’s instructions, and then reverse-transcribed using TransScript One-Step gDNA Removal and cDNA Synthesis SuperMix (TransGen, AT311, Beijing, China). The cDNA was diluted 10 times with sterilized distilled water. Quantitative RT-PCR (qRT-PCR) was conducted, according to the instructions of TB Green Premix Ex Taq II (Tli RNaseH Plus) (Takara, RR820A, Dalian, China), on a ABI QuantStuio 7 Flex RT-PCR instrument (Applied Biosystems, Foster City, CA, USA). Gene-specific primers for qRT-PCR were shown in Table S3. The relative expression levels of the target *CCT* genes were presented as fold-change, calculated using the comparative method.

## Figures and Tables

**Table 1:** Properties and locations of the predicted CCT (CO, COL and TOC1) proteins in *M. truncatula*.

Gene	Chromosome	CDS (Coding Sequence)	Protein (aa)	Molecular Weight (D)	Theoretical Pi	Subfamily
*MtCCT1*	Chr1	Medtr1g008220.1	282	31927	4.94	CMF
*MtCCT2*	Chr1	Medtr1g013450.1	316	35496	6.31	COL
*MtCCT3*	Chr1	Medtr1g044785.1	230	25360	5.6	CMF
*MtCCT4*	Chr1	Medtr1g067110.1	748	81610	7.27	PRR
*MtCCT5*	Chr1	Medtr1g073350.1	414	45719	4.47	CMF
*MtCCT6*	Chr1	Medtr1g110870.1	436	48793	5.45	COL
*MtCCT7*	Chr2	Medtr2g068730.1	266	30816	5.21	CMF
*MtCCT8*	Chr2	Medtr2g088900.1	521	57571	5.85	COL
*MtCCT9*	Chr2	Medtr2g096080.1	251	28657	6.17	CMF
*MtCCT10*	Chr3	Medtr3g037390.1	575	64621	6.05	PRR
*MtCCT11*	Chr3	Medtr3g082630.2	411	45277	4.89	COL
*MtCCT12*	Chr3	Medtr3g091340.1	281	31916	5.23	CMF
*MtCCT13*	Chr3	Medtr3g092780.1	685	75851	6.11	PRR
*MtCCT14*	Chr3	Medtr3g100040.1	359	38847	4.47	CMF
*MtCCT15*	Chr3	Medtr3g100050.1	309	34206	5.62	CMF
*MtCCT16*	Chr3	Medtr3g105710.1	290	31869	7.11	COL
*MtCCT17*	Chr4	Medtr4g008090.1	121	13589	9.86	CMF
*MtCCT18*	Chr4	Medtr4g061360.1	796	87246	6.72	PRR
*MtCCT19*	Chr4	Medtr4g061823.1	242	27580	5.93	CMF
*MtCCT20*	Chr4	Medtr4g061910.1	242	27580	5.93	CMF
*MtCCT21*	Chr4	Medtr4g093730.1	334	37563	5.04	CMF
*MtCCT22*	Chr4	Medtr4g108880.1	585	68781	5.80	PRR
*MtCCT23*	Chr4	Medtr4g127420.1	379	42006	9.00	CMF
*MtCCT24*	Chr4	Medtr4g128930.1	375	41119	5.86	COL
*MtCCT25*	Chr5	Medtr5g010120.1	251	29195	5.83	CMF
*MtCCT26*	Chr5	Medtr5g066510.1	286	31424	5.53	CMF
*MtCCT27*	Chr5	Medtr5g069480.1	410	45027	5.70	COL
*MtCCT28*	Chr5	Medtr5g072780.1	217	25897	5.07	CMF
*MtCCT29*	Chr7	Medtr7g018170.1	396	43757	5.10	COL
*MtCCT30*	Chr7	Medtr7g032240.1	352	40433	9.53	CMF
*MtCCT31*	Chr7	Medtr7g083540.1	390	44311	6.28	COL
*MtCCT32*	Chr7	Medtr7g108150.1	372	42581	6.21	COL
*MtCCT33*	Chr7	Medtr7g118260.1	596	66654	6.54	PRR
*MtCCT34*	Chr8	Medtr8g024260.1	640	73001	6.74	PRR
*MtCCT35*	Chr8	Medtr8g098725.1	324	37326	5.34	CMF
*MtCCT36*	Chr8	Medtr8g104190.1	416	47041	5.10	COL

## Supplementary Materials

The following are available online at https://www.mdpi.com/2223-7747/9/4/513/s1, Table S1: The orthologous *CCT* gene pairs between *M. truncatula* and *Arabidopsis thaliana* /*Oryza sativa*., Table S2: Detailed expression levels of *MtCCT* genes in six tissues, developing seed/nodule and treatments in response to different abiotic stresses and hormones., Table S3: Premier sequences used for qRT-PCR in this study., Figure S1: Alignment of multiple MtCCT protein sequences., Figure S2: Sequence information of CCT, BBOX and REC domains in MtCCT proteins., Figure S3: Chromosomal location of *MtCCT* genes in *M. truncatula* genome., Figure S4: The expression heatmaps of 16 *MtCCT* genes during the development of seeds (a) and nodules (b)., Figure S5: Distribution of the *cis*-elements responsive to light and phytohormones in the promoter regions of *MtCCT* genes.
