# Supplementary material for: Genome-Wide Identification, Expression Analysis and Functional Study of CCT Gene Family in Medicago truncatula
Source: Plants (Basel). 2020 Apr 16;9(4):513. doi: 10.3390/plants9040513 (PMC7238248; doi:10.3390/plants9040513)
Supplement: Supplementary file 1 [file plants-09-00513-s001.zip › Supplementary materials/Table S1.docx]

**Table S1** The orthologous *CCT* gene pairs between *M. truncatula* and *Arabidopsis thaliana* /*Oryza sativa*.

| ***Medicago truncatula*** | ***Oryza sativa*** | ***Arabidopsis thaliana*** |
| --- | --- | --- |
| *MtCCT1* |  | *AT4G27900.1* |
| *MtCCT2* |  | *AT2G24790.1* |
| *MtCCT3* |  | *AT2G33350.5* |
| *MtCCT4* | *Os03g0284100* | *AT5G02810.1* |
| *MtCCT5* |  | *AT2G33350.5* |
| *MtCCT6* |  | *AT1G68520.1* |
| *MtCCT7* |  | *AT3G12890.1* |
| *MtCCT8* |  | *AT1G05290.1* |
| *MtCCT9* | *Os01g0835700* |  |
| *MtCCT10* |  |  |
| *MtCCT11* |  |  |
| *MtCCT12* |  | *AT4G27900.1* |
| *MtCCT13* |  | *AT2G46790.1* |
| *MtCCT14* |  | *AT4G24470.3* |
| *MtCCT15* |  |  |
| *MtCCT16* |  | *AT2G24790.1* |
| *MtCCT17* |  |  |
| *MtCCT18* | *Os03g0284100* | *AT5G60100.2* |
| *MtCCT19* |  | *AT5G59990.1* |
| *MtCCT20* |  |  |
| *MtCCT21* |  |  |
| *MtCCT22* |  | *AT5G61380.1* |
| *MtCCT23* | *Os06g0699600* | *AT5G57180.2* |
| *MtCCT24* | *Os02g0178100* | *AT5G57660.1* |
| *MtCCT25* |  | *AT1G63820.1* |
| *MtCCT26* |  | *AT4G24470.3* |
| *MtCCT27* |  |  |
| *MtCCT28* |  |  |
| *MtCCT29* |  |  |
| *MtCCT30* |  | *AT5G14370.1* |
| *MtCCT31* |  |  |
| *MtCCT32* | *Os03g0351100* | *AT2G47890.1* |
| *MtCCT33* |  | *AT2G46790.1* |
| *MtCCT34* |  | *AT2G46790.1* |
| *MtCCT35* |  |  |
| *MtCCT36* |  |  |
