# Supplementary material for: Genome-Wide Identification, Expression Analysis and Functional Study of CCT Gene Family in Medicago truncatula
Source: Plants (Basel). 2020 Apr 16;9(4):513. doi: 10.3390/plants9040513 (PMC7238248; doi:10.3390/plants9040513)
Supplement: Supplementary file 1 [file plants-09-00513-s001.zip › Supplementary materials/Table S3.docx]

**Table S3** Premier sequences used for qRT-PCR in this study.

| **Target** | **Forward sequence** | **Reverse sequence** |
| --- | --- | --- |
| *MtCCT1* | TGCGGAGAGCGTTTAGTGAAG | TTCCTCGGATTCTTGGTTGACTG |
| *MtCCT2* | CCTCACCACCACCTTCAACC | CGGAACGACGCCATCTGC |
| *MtCCT3* | GCGAGTTAGAGGAAGGTTTGC | TCAATGAGTGGATGGAATAGTTGC |
| *MtCCT4* | ATGAGGAGTTGCCATCTCTTGAG | TGAGTTCTGGTTAGGAGGATTGC |
| *MtCCT5* | CAGTCACATCATCACAGCAAGTAG | AGAAGGAGAAGAAGGGTTCAAAGG |
| *MtCCT6* | TTTCACGGGCTTCTTCCATCTG | GTTCTCCACACTTCTCCTTCACC |
| *MtCCT7* | TGGGATTTTATGGTGGAGGGTTTC | GTTTGGTTCTTCAACTGCTGCTAG |
| *MtCCT8* | AAGTTGGGCGAAGGAGATACG | TGTGGCAGTTTGAGAGCAGTC |
| *MtCCT9* | CTCAACACCCTTTCTTCTTCACTC | AATGACTGCTAATGCTCCTTTGC |
| *MtCCT10* | ATGTCCTTCCCGTATTACCCAATG | CCGTCTTTCAGCAAGTTGTTTCC |
| *MtCCT11* | GGTGGTGCTGGAGACTATCAAG | GCTCAAAGGGTCATAATCATAGGC |
| *MtCCT12* | AGCCTGCTTTCCTTGGTATCTC | TCCTCCCGAAATTCCTCTTTGTC |
| *MtCCT13* | CACAATCAGGTCCACCATCAATG | GCCACTACTTCCACAATTACTTCC |
| *MtCCT14* | GCCAACAAGGGGACACTCAG | CACGGGTTCCTGCTCATCATC |
| *MtCCT15* | GTTTGGACACATCGCCTCTAAATC | TGAACTACATCTTGCCCTGACTC |
| *MtCCT16* | AGAGTTAGAAACACCGTCACAGTC | TTTCGCAAACCTCCCTTTAATCC |
| *MtCCT17* | ATTTAGCGTCCCTCAGAGATTAGC | AGCCAGAGCCAGAAGATTATCAAG |
| *MtCCT18* | GCACACTCACCATCAACTTCAAC | GCTTCCACCCTTTCCATTACTCC |
| *MtCCT19/20* | TTACAGCCCAGAGGAAAAGAAGG | GGAGCAAGATTTGAAGTAGGAACC |
| *MtCCT21* | CGGCTGGTCCAAGAACTCTATG | CAGGTGACTGTCTGGCATCC |
| *MtCCT22* | CGGTCAGGTTTCTACAGTTCATC | ATCGTCATAGTCGGTGGAAGC |
| *MtCCT23* | TACAGAGAGGTGGAGTGAGAGC | CGCCGTCGTAATTCAATTTCAAC |
| *MtCCT24* | GATTCCACCATTCACTCTGCTAAC | CCGTAACCGTCGTCAGTAGG |
| *MtCCT25* | CACCTGGGTCTCAAACGGATG | GATTGTCTGCTAGTGTCTTTCGG |
| *MtCCT26* | ACGCTCAACATCAAATTCAATACG | CCGCATTCACACCAACATTAGG |
| *MtCCT27* | CCGCTCTGATGCTGCTTCC | TGATTGATTGCCTCTTGTGTGTTG |
| *MtCCT28* | AGTGGATGATGAAAAGAGTTTGGC | CAAAGCGACCCTTGATTCTTGG |
| *MtCCT29* | ACCACCACCTCAGCCACAG | GCAAAGAGAAGCAGCATCAGC |
| *MtCCT30* | GCTCATCATCACGATACAGAACAC | TCATTCCTCTTCCTCCTCATTGC |
| *MtCCT31* | GGAAGTTGCGAAGAGGAAGAGG | GGAGGTTGATACGACGGATTACG |
| *MtCCT32* | ACGCCTTCAGAACCTCTCAAC | CTCTCCGCCCGAACTTTACG |
| *MtCCT33* | TTGGTCAGTCTGGGCAAGTTG | ACAAGTATAGGTGGCATCATCCG |
| *MtCCT34* | TTCCTCCTCCCAGTCCAATCC | CCGCATTTGAAATAGCCTCTCC |
| *MtCCT35* | AAGTAGGTCGTTATAGTGCGGAAG | CTCTGTGGGCTCGTCATTGC |
| *MtCCT36* | TAAGGTAACAACAACAGCACAAGC | TGGCACACGACACAGCAAC |
| *MtACTIN* | GCTGACCGTATGAGCAAGGA | TGCCAAGATAGACCCACCAA |
