# Supplementary figures and images for: Genome-Wide Identification, Expression Analysis and Functional Study of CCT Gene Family in Medicago truncatula
Source: Plants (Basel). 2020 Apr 16;9(4):513. doi: 10.3390/plants9040513 (PMC7238248; doi:10.3390/plants9040513)

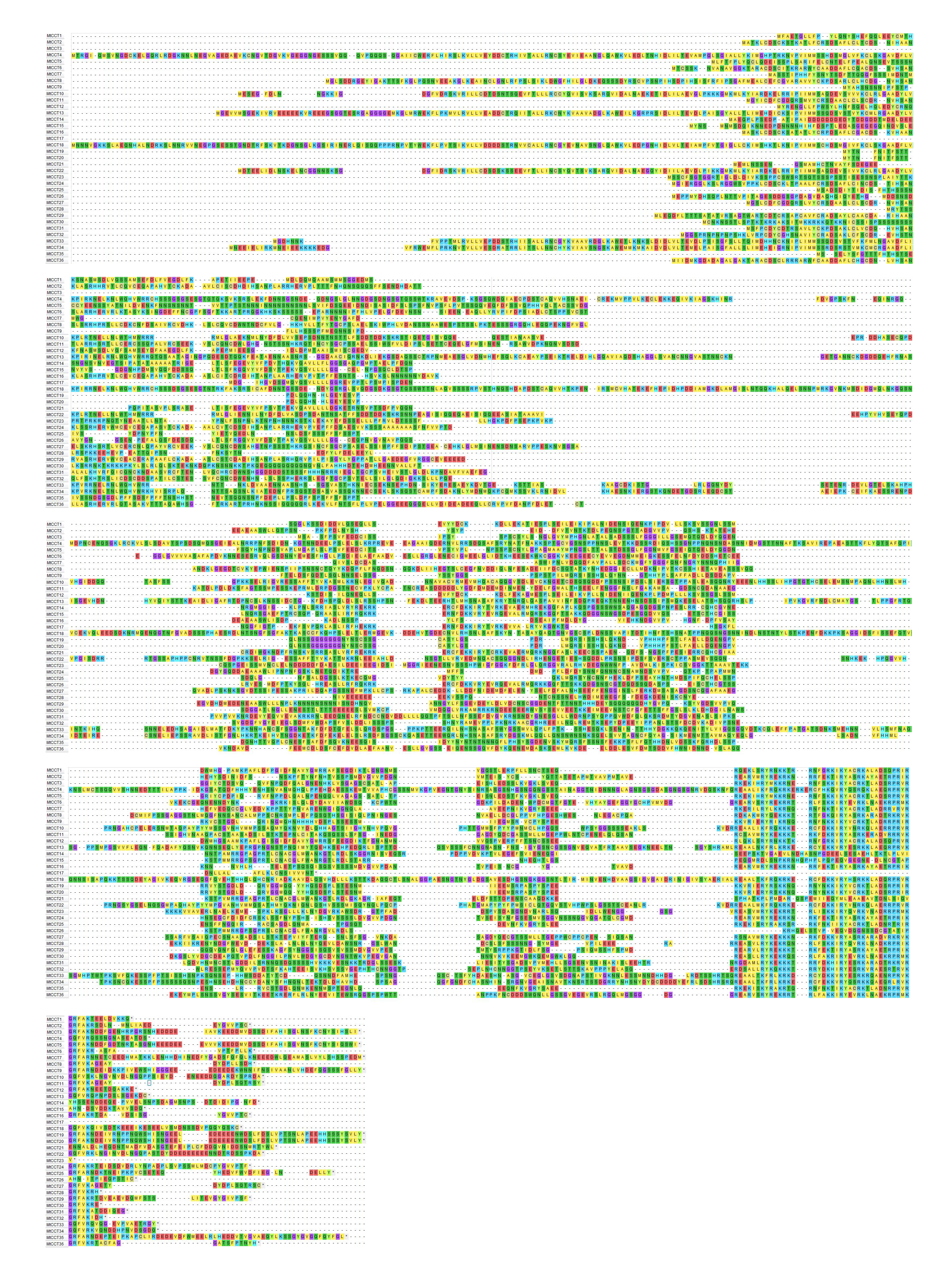

Supplement: Supplementary file 1 [file plants-09-00513-s001.zip › Supplementary materials/Figure S1.tif]

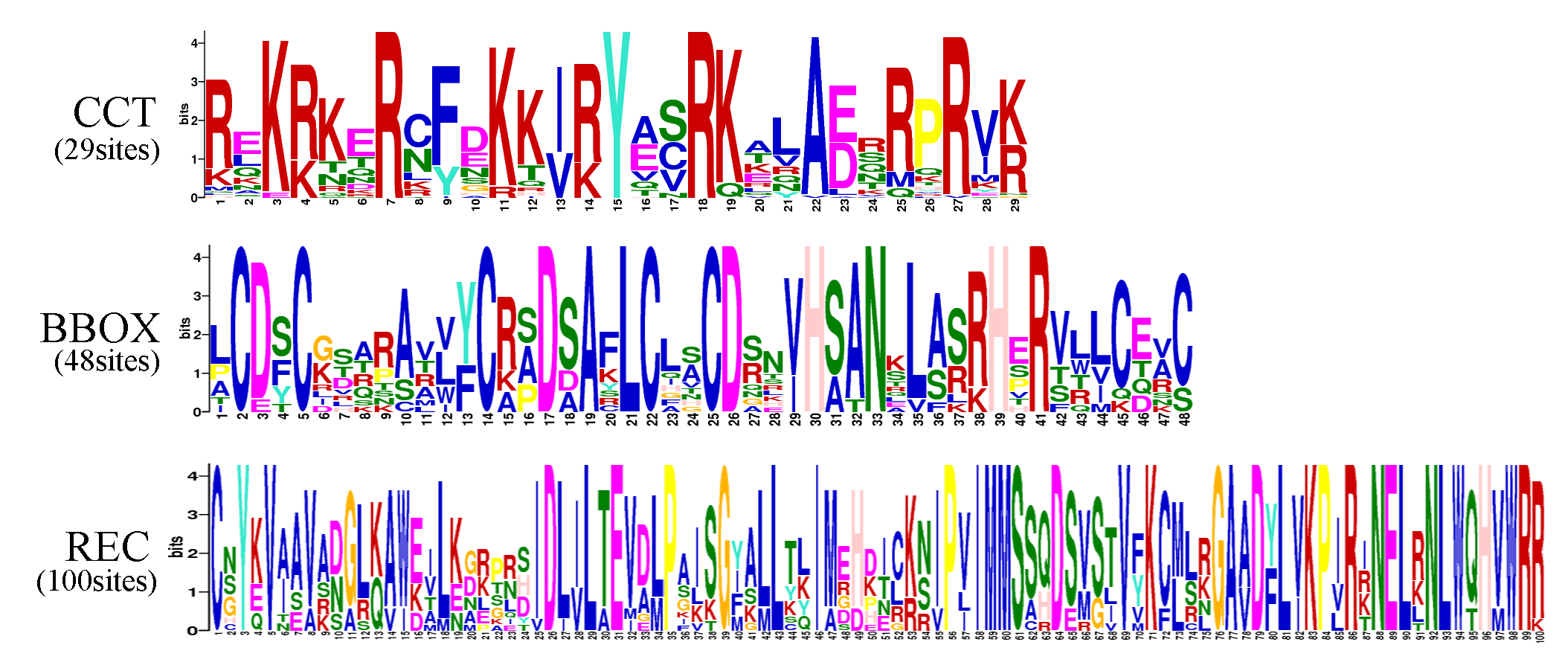

Supplement: Supplementary file 1 [file plants-09-00513-s001.zip › Supplementary materials/Figure S2.tif]

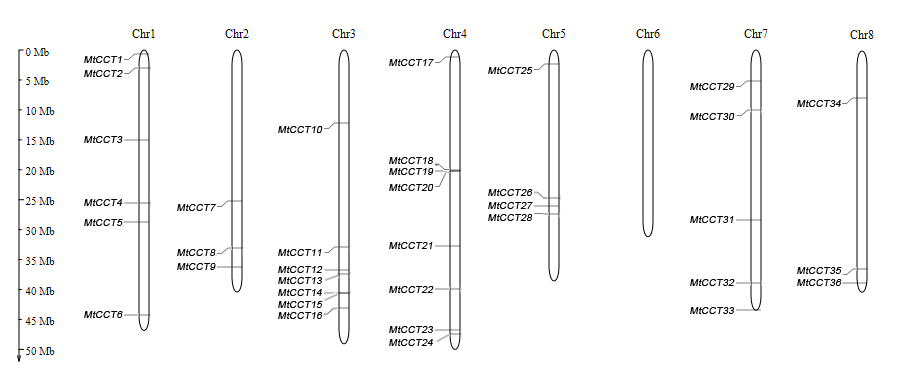

Supplement: Supplementary file 1 [file plants-09-00513-s001.zip › Supplementary materials/Figure S3.tif]

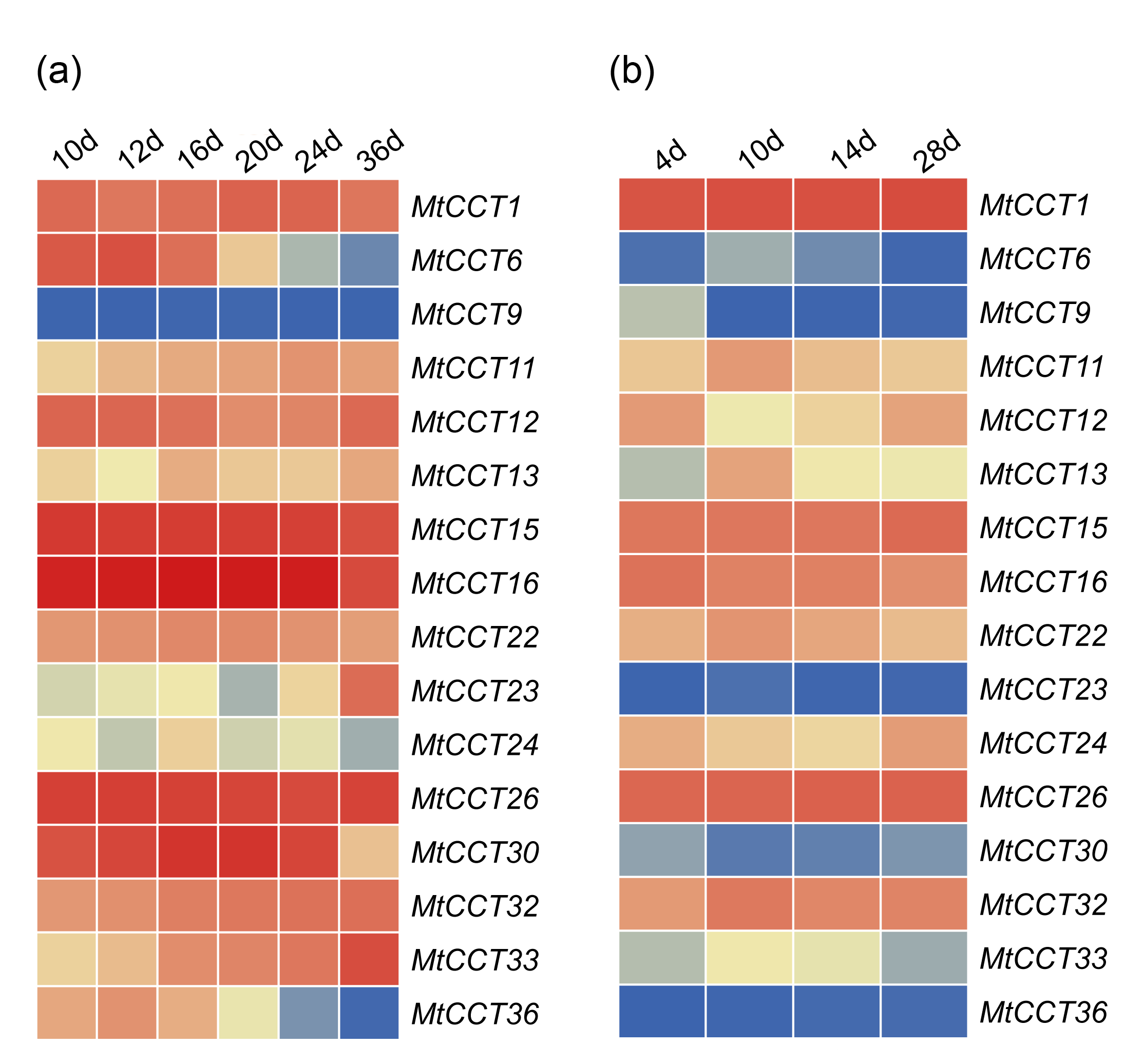

Supplement: Supplementary file 1 [file plants-09-00513-s001.zip › Supplementary materials/Figure S4.tif]

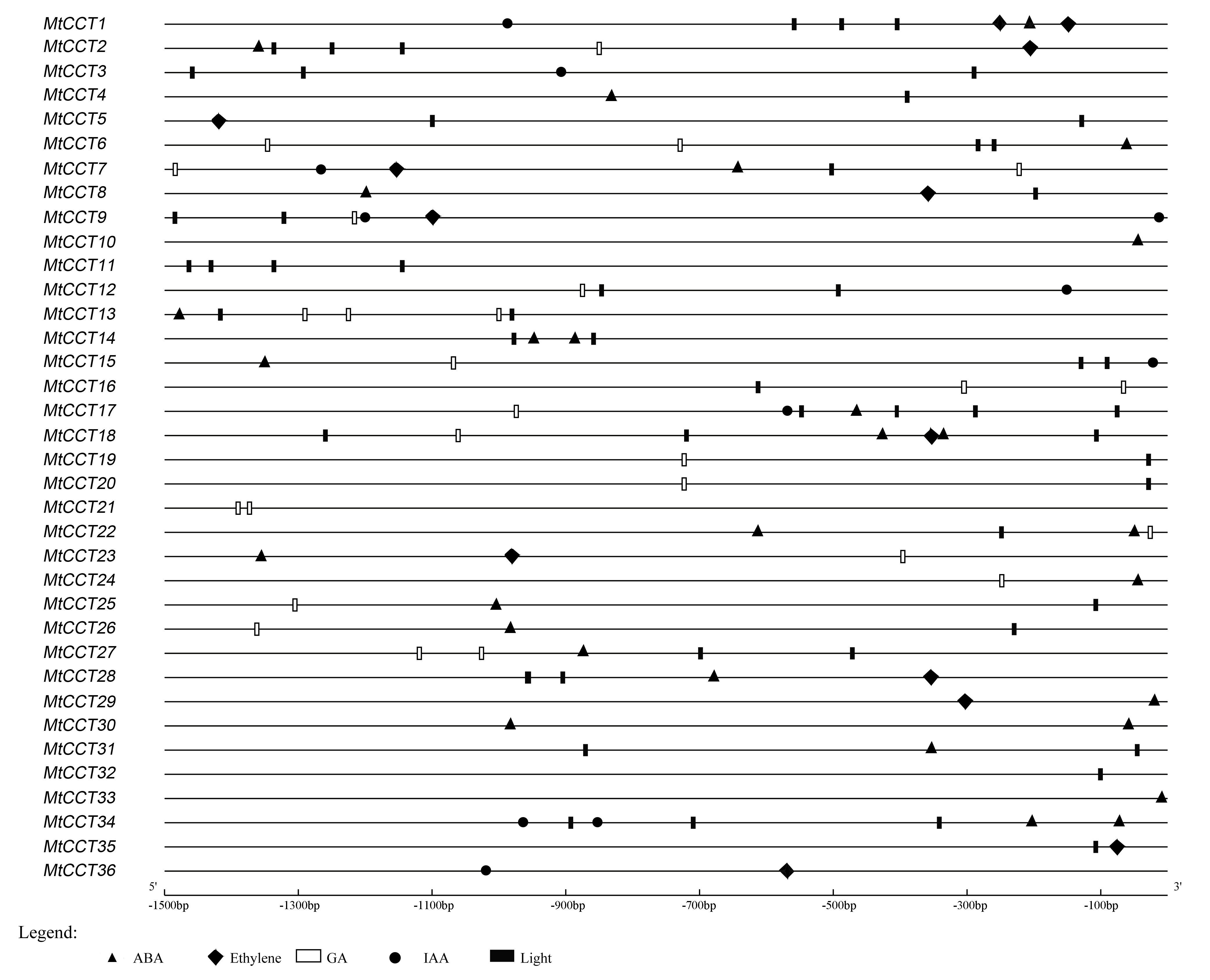

Supplement: Supplementary file 1 [file plants-09-00513-s001.zip › Supplementary materials/Figure S5.tif]
